# Enhancing Diagnostic Accuracy and Procedural Outcomes in Interventional Cardiology Through Machine Learning Algorithms

**DOI:** 10.1016/j.jscai.2024.102511

**Published:** 2025-03-18

**Authors:** Zain Khalpey, Amina Khalpey, Saurabh Jha

**Affiliations:** a HonorHealth, Scottsdale, Arizona; b Khalpey AI Lab, Scottsdale, Arizona; c Applied Translational Artificial Research Institute (ATARI), Scottsdale, Arizona; d Department of Radiology, University of Pennsylvania, Philadelphia, Pennsylvania

**Keywords:** clinical integration, diagnostic accuracy, ethical implementation, interventional cardiology, machine learning, procedural outcomes

## Introduction

In the rapidly evolving landscape of interventional cardiology, the advent of artificial intelligence (AI) and machine learning (ML) heralds a transformative era. Interventional cardiologists are now at the cusp of a significant shift in their practice, driven by 2 pivotal trends. First, AI's capability to uncover more subclinical diseases is enhancing the diagnostic value of intravascular imaging modalities such as intravascular ultrasound (IVUS) and optical coherence tomography (OCT), improving preprocedural planning for complex coronary and structural interventions.^1^ Second, AI is revolutionizing periprocedural risk stratification, leading to more personalized approaches in interventional decision-making, particularly in high-risk percutaneous coronary intervention (PCI), transcatheter aortic valve replacement (TAVR), and congenital heart interventions.^2^

## The inadequacies of current practices

Current interventional cardiology practices, including coronary angiography interpretation, intravascular imaging analysis, and fractional flow reserve (FFR) assessment, rely heavily on operator expertise. However, studies show significant interobserver variability in these critical assessments.^3^ This subjectivity can result in unnecessary procedures, suboptimal stent placement, or missed opportunities for intervention, potentially impacting patient outcomes and health care costs.^4^

For instance, a multicenter study found only 80% agreement between interventional cardiologists on the need for revascularization based on coronary angiography alone. Similarly, the interpretation of IVUS images for stent sizing and positioning can vary significantly between operators, affecting procedural success rates. In structural heart interventions, such as TAVR, variability in annular sizing can lead to suboptimal valve selection and an increased risk of paravalvular leak.

Although operator judgment remains indispensable, ML algorithms can substantially boost the consistency and accuracy of these critical assessments in the catheterization laboratory.

## ML—A powerful tool for transformation

In interventional cardiology, ML can analyze vast amounts of angiographic images, intravascular imaging data, hemodynamic parameters, and clinical variables to enhance decision-making and procedural outcomes.^5^ Specifically, 4 high-impact areas hold promise:

1. Automated angiographic and intravascular imaging analysis: ML can enable automated analysis of coronary angiograms, IVUS, and OCT images, providing rapid, objective assessments of lesion characteristics, vessel dimensions, and optimal stent landing zones. For instance, deep learning algorithms have shown promise in automatically detecting and quantifying coronary stenoses from angiographic images, potentially reducing interobserver variability.
2. Personalized procedural risk prediction: ML algorithms can assimilate patient-specific factors, lesion characteristics, and procedural variables to predict the risk of periprocedural complications. Novel ML-based risk scores have demonstrated improved performance in predicting adverse events after PCI compared to traditional risk models.
3. Intraprocedural decision support: ML models can offer real-time recommendations during interventions by analyzing live angiographic and intravascular imaging data. AI-powered platforms are being developed to provide guidance for stent sizing and positioning based on the fusion of angiographic and OCT data.
4. Outcome prediction and quality improvement: Supervised learning algorithms can analyze postprocedural data to predict long-term outcomes and identify areas for quality improvement. Recent studies have demonstrated that ML models could predict 1-year target lesion failure after PCI with greater accuracy than traditional risk scores.

Several institutions have begun integrating ML-powered tools into their interventional cardiology practices. For example, ML-powered FFR-CT analysis has been integrated into preprocedural planning workflows for complex PCI cases in some centers, reducing the need for invasive FFR measurements and improving procedural efficiency.^6^

Artificial intelligence–powered IVUS analysis tools are being implemented to guide stent sizing and positioning in left main coronary artery interventions, with early results showing promise in reducing stent undersizing and improving long-term outcomes compared to conventional IVUS interpretation.

In the realm of structural heart interventions, AI platforms for automating preprocedural CT analysis for TAVR sizing have shown excellent correlation with expert measurements while significantly reducing analysis time.

These examples underscore ML's maturity for real-world adoption in the catheterization laboratory. However, widespread success necessitates interventional cardiologist training in navigating these tools confidently and efficiently.

## Future outlook

The future of ML in interventional cardiology is poised to redefine patient care across the entire spectrum of coronary, structural, and congenital interventions^7^:

• Predictive analytics for device selection: ML algorithms could optimize device selection in complex PCI and structural interventions by analyzing patient-specific anatomical and physiological data.
• Real-time procedural guidance: Advanced ML models could provide real-time, holographic guidance for complex interventions, such as chronic total occlusion PCI or structural heart procedures, by fusing preprocedural imaging with live fluoroscopy.
• Personalized postprocedural care: ML-driven analysis of procedural data, combined with patient-specific factors, could tailor postintervention antiplatelet therapy and follow-up protocols.

Through such advances, ML promises to enhance the precision and personalization of interventional cardiology, potentially improving both short-term procedural success and long-term patient outcomes.

## Progressing responsibly

Despite the enormous promise, ML also introduces concerns regarding explainability and bias.^8^ Cardiology AI models typically comprise neural networks processing thousands of variables—essentially impenetrable “black boxes” making it hard to understand reasons behind individual predictions. However, techniques like explainable AI help attribution mapping and model simplification to deconstruct these black boxes.^9^

If source data itself have demographic or geographic biases, the models can propagate these inequalities through recommendations exacerbating disparities. However, diverse training data representative of real-world populations can mitigate this.

Addressing such factors through ongoing research and governance frameworks for responsible AI will be vital as these technologies become entrenched in clinical practice.

## Advancing the backend architecture

The predictive capabilities and real-world value of any ML system fundamentally depend on the robustness of its underlying data infrastructure and algorithms. Here, innovations on multiple fronts are enhancing the reliability and trustworthiness of interventional cardiology–focused AI^10^:

1. Federating distributed data: To improve model generalizability beyond individual health systems, platforms are being developed to enable decentralized, privacy-preserving data sharing across institutions for collective ML without moving data. Such federated learning on diverse, multisite data can enhance model performance and curb bias.
2. Quantum ML: Emerging quantum computing hardware promises to massively scale up ML model complexity allowing assimilation of countless physiological, cellular, and environmental variables spanning decades per patient. Researchers are exploring quantum algorithms for various cardiology applications, including electrocardiogram classification and revascularization needs prediction.
3. Large language models: Foundation models like GPT-3 point toward AI's future. Cardiology-focused large language models pretrained on millions of patient records, trial data, biomedical journals, and guidelines could gain human-like comprehension to provide reasoned, referenced recommendations. Still, responsible development guarding against false confidence in automation will be vital.
4. Trust and verification architectures: To ensure model accountability, innovative validation schemas are being developed to allow continuous auditability regarding model-recommended interventions against actual outcomes to detect any reliability drifts and reveal biases. Integrating such governance can bolster physician and public trust in AI.

At the same time, these tools do not displace but rather augment clinicians. Human oversight remains essential for nuanced considerations in complex cardiac care beyond ML's current capabilities. Ultimately through human-AI synthesis, interventional cardiology aims to elevate patient benefit to new heights.

## Key challenges in ML for interventional cardiology

**Figure 1:**
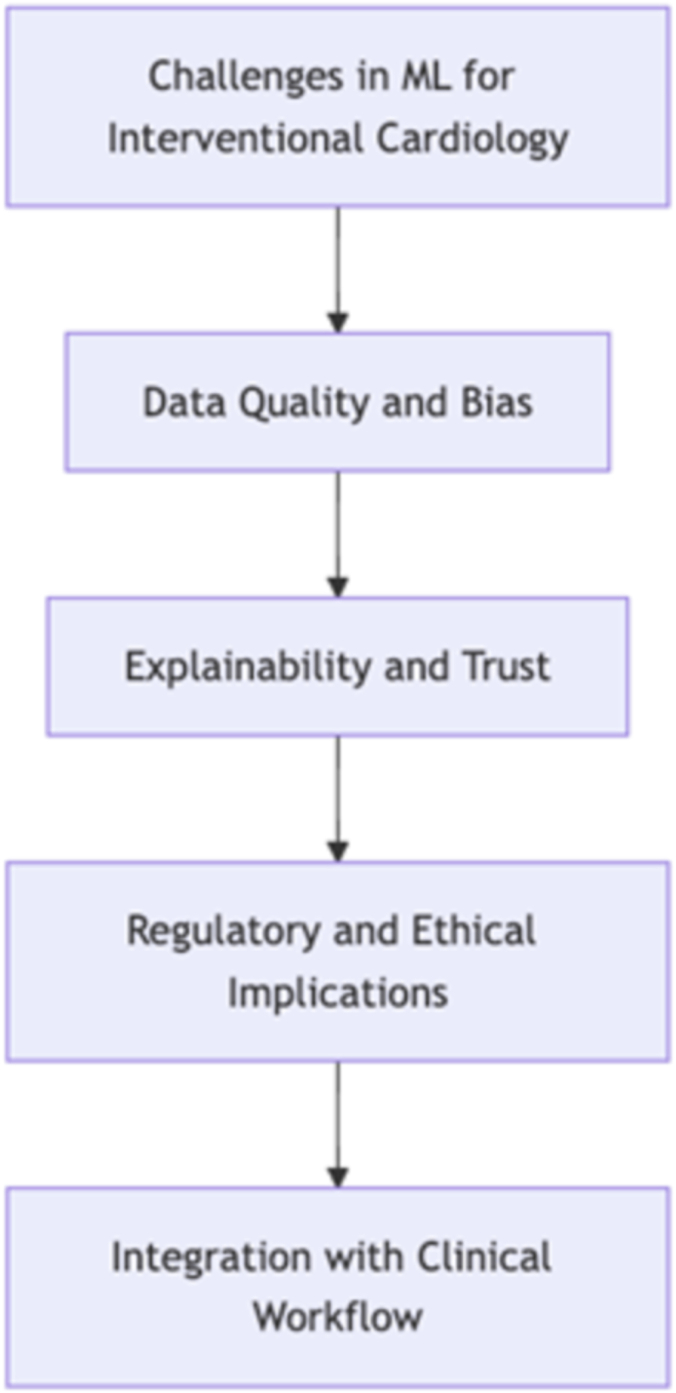
**A flowchart showing the Challenges in ML for Interventional Cardiology.** ML, machine learning.

Although the potential of ML in interventional cardiology is significant, there are several key challenges that need to be addressed for successful implementation. These challenges can be visualized in a hierarchical structure as follows (Figure 1):

1. Data quality and bias: Ensuring the quality, representativeness, and unbiased nature of the data used to train ML models is crucial. This includes addressing issues of data variability, missing data, and potential biases in data collection that could lead to skewed results.
2. Explainability and trust: As ML models become more complex, ensuring their decisions are interpretable and explainable to clinicians becomes increasingly important. This is crucial for building trust in ML systems and their integration into clinical decision-making processes.
3. Regulatory and ethical implications: The use of ML in health care raises important regulatory and ethical questions. These include issues of patient privacy, data ownership, liability in case of errors, and ensuring equitable access to ML-enhanced care.
4. Integration with clinical workflow: Successfully incorporating ML tools into existing clinical workflows without disrupting patient care or overburdening health care providers is a significant challenge. This requires careful design of user interfaces, training of clinical staff, and seamless integration with existing hospital information systems.

Addressing these challenges will be crucial for the successful and responsible implementation of ML in interventional cardiology. It will require collaboration between clinicians, data scientists, ethicists, and regulatory bodies to ensure that ML enhances patient care while maintaining the highest standards of safety and ethical practice.

## Conclusion

ML promises a new era for patient care in interventional cardiology. As specialists witnessing its accelerating adoption in catheterization laboratories worldwide, we believe ML has reached an inflection point in technological maturity and demonstrated clinical value to graduate from promise to routine integration.^11^ Indeed, it holds the disruptive power to make preprocedural planning more precise, intraprocedural decision-making more informed, and postprocedural care more personalized. To fully unlock this transformation, all stakeholders—interventional cardiologists, hospitals, medical device companies, and technology innovators—must align to responsibly harness the power of human-machine collaboration in the catheterization laboratory.
